# Association of non-steroidal anti-inflammatory medications and aspirin with colorectal cancer incidence in older adults

**DOI:** 10.1093/jnci/djaf145

**Published:** 2025-06-14

**Authors:** Farzana Y Zaman, Suzanne G Orchard, Galina Polekhina, Peter Gibbs, Wendy B Bernstein, Finlay Macrae, Jeanne Tie, Jeremy Millar, Lucy Gately, Luz María Rodríguez, Gijsberta J van Londen, Victoria Mar, Emma Hiscutt, Nikki Adler, Aaron Kent, Wee Loon Ong, Andrew Haydon, Erica Warner, Andrew T Chan, John Zalcberg

**Affiliations:** Department of Medical Oncology, Alfred Health, Melbourne, VIC, Australia; School of Public Health and Preventive Medicine, Monash University, Melbourne, VIC, Australia; School of Public Health and Preventive Medicine, Monash University, Melbourne, VIC, Australia; Personalised Oncology Division, Walter and Eliza Hall Institute for Medical Research, Melbourne, VIC, Australia; Department of Medical Oncology, Peter MacCallum Cancer Centre, Melbourne, VIC, Australia; Department of Medical Oncology, Walter Reed National Military Medical Center, Bethesda, MD, United States; Uniformed Services University of Health Sciences, F E Hebert School of Medicine, Bethesda, MD, United States; Department of Colorectal Medicine and Genetics, The Royal Melbourne Hospital, Melbourne, VIC, Australia; Personalised Oncology Division, Walter and Eliza Hall Institute for Medical Research, Melbourne, VIC, Australia; Department of Medical Oncology, Peter MacCallum Cancer Centre, Melbourne, VIC, Australia; Sir Peter MacCallum Department of Oncology, University of Melbourne, Melbourne, VIC, Australia; School of Public Health and Preventive Medicine, Monash University, Melbourne, VIC, Australia; Department of Medical Oncology, Alfred Health, Melbourne, VIC, Australia; Personalised Oncology Division, Walter and Eliza Hall Institute for Medical Research, Melbourne, VIC, Australia; Division of Cancer Prevention, National Cancer Institute, Bethesda, MD, United States; Department of Surgery, Walter Reed National Military Medical Center, Bethesda, MD, United States; Division of Cancer Prevention, National Cancer Institute, Bethesda, MD, United States; School of Public Health and Preventive Medicine, Monash University, Melbourne, VIC, Australia; Victorian Melanoma Service, The Alfred Hospital, Melbourne, VIC, Australia; School of Public Health and Preventive Medicine, Monash University, Melbourne, VIC, Australia; Victorian Melanoma Service, The Alfred Hospital, Melbourne, VIC, Australia; School of Public Health and Preventive Medicine, Monash University, Melbourne, VIC, Australia; Victorian Melanoma Service, The Alfred Hospital, Melbourne, VIC, Australia; Department of Radiation Oncology, Alfred Health, Melbourne, VIC, Australia; School of Public Health and Preventive Medicine, Monash University, Melbourne, VIC, Australia; Department of Radiation Oncology, Alfred Health, Melbourne, VIC, Australia; Department of Medical Oncology, Alfred Health, Melbourne, VIC, Australia; School of Public Health and Preventive Medicine, Monash University, Melbourne, VIC, Australia; Clinical and Translational Epidemiology Unit, Massachusetts General Hospital and Harvard Medical School, Boston, MA, United States; Clinical and Translational Epidemiology Unit, Massachusetts General Hospital and Harvard Medical School, Boston, MA, United States; Department of Medical Oncology, Alfred Health, Melbourne, VIC, Australia; School of Public Health and Preventive Medicine, Monash University, Melbourne, VIC, Australia

## Abstract

**Background:**

The relationship between aspirin, and/or other non-steroidal anti-inflammatory drugs (NSAIDs), and colorectal cancer (CRC) risk in older adults is uncertain. This study investigated the association between non-aspirin NSAIDs (NA-NSAIDs) use, alone or combined with aspirin, on CRC incidence in older adults.

**Methods:**

This is a post hoc analysis of ASPirin in Reducing Events in the Elderly (ASPREE) randomized controlled trial data and its observational continuation, ASPREE-XT (median follow-up, 8.4 years [IQR: 7.2-9.6]). NA-NSAID exposure was ascertained by self-report and medical record review at baseline, for all ASPREE participants, and for Australian participants, via linkage to the Pharmaceutical Benefits Scheme (PBS). CRC was an adjudicated secondary endpoint of ASPREE. We investigated the association between NA-NSAID use alone, and in combination with randomized aspirin use, on the incidence of CRC in time-to-event analyses.

**Results:**

Of 19 114 ASPREE participants, 2713 (14%) reported NA-NSAID use at baseline. NA-NSAID use was associated with a reduced incidence of CRC (HR^NA-NSAID use: Yes vs No^ = 0.74; 95% CI = 0.56 to 0.98). This association between NA-NSAIDs and CRC was not modified by aspirin (*P*-value for interaction term of 0.81). When assessing NA-NSAID use over 2 years post-randomization in Australian participants who consented to the use of PBS data (*n* = 13 725), a similar reduction in CRC risk was observed (HR^High NA-NSAID use vs None^ = 0.52, 95% CI = 0.32 to 0.83).

**Conclusions:**

NA-NSAID use in Australian and American adults over the age of 70 years was associated with a reduced CRC incidence, which increased with increasing exposure. Aspirin did not modify the effect of NA-NSAIDs on CRC incidence.

## Introduction

The ASPirin in Reducing Events in the Elderly (ASPREE) trial was a randomized, placebo-controlled trial (RCT) which explored whether daily low-dose aspirin (100 mg), vs placebo, prolonged disability-free survival in 19 114 otherwise healthy Australian and American adults aged 70+ years (65+ years for American minorities). Colorectal cancer (CRC) was the most common cancer diagnosis, excluding the sex-specific cancers (prostate and breast cancer), documented during the ASPREE RCT. No effect of aspirin on CRC incidence was detected (HR Aspirin vs Placebo = 1.02; 95% CI = 0.81 to 1.30).^1^

These findings with respect to CRC incidence are in contrast to previously published studies involving the use of aspirin in cancer prevention, generally conducted in cohorts of middle-aged and/or high-risk individuals, demonstrating reduced cancer incidence and mortality, particularly for CRC, with aspirin use.^2-4^ Systematic reviews generally conclude that aspirin use is associated with a decreased incidence of colonic adenomas,^5^ CRCs and CRC-related mortality.^2^^,^^6^

There is mixed evidence for the role of non-aspirin non-steroidal anti-inflammatory drugs (NA-NSAIDs) in mitigating CRC incidence, with some population-based studies reporting that NA-NSAIDs are associated with a reduction in CRC risk,^5^^,^^7-9^ while other studies only showed benefits in particular high-risk subgroups.^10^^,^^11^ Systematic reviews of RCTs have concluded that NA-NSAIDs, such as COX 2-inhibitors, are effective in preventing metachronous neoplasia or adenoma recurrence, in patients with a high baseline risk of CRC.^5^^,^^12^ Notably, however, there is limited data in older populations, a group which has higher prevalence of NA-NSAID use.

Previous studies estimate that up to 27% of regular aspirin users simultaneously take NA-NSAIDs.^13^ Based on clinical trials pointing to the apparent benefits of aspirin (albeit not seen in the ASPREE RCT) and the potential reduction in CRC associated with the use of NA-NSAIDs alone in prior studies,^14^ we hypothesized that combined use of NA-NSAIDs and aspirin may have had a synergistic effect. Limited data exists on the combined use of NA-NSAIDs and aspirin in CRC risk, particularly in older populations. This study sets out to explore the impact of NA-NSAID use alone, and in combination with aspirin, on CRC incidence in older adults, utilizing data from the ASPREE RCT and the extended observational follow-on (ASPREE-XT).

## Methods

### Study design

Details of the ASPREE trial have previously been published, along with the main cancer outcomes.^1^^,^^15^ Briefly, between March 2010 and December 2014, a total of 19 114 Australian (*n* = 16 703, aged 70+ years) and American (*n* = 2411, aged 65+ years) participants were randomly assigned to receive daily low-dose aspirin 100 mg (*n* = 9525) or matching placebo (*n* = 9589). Participants were required to be free of cardiovascular disease, dementia, and independence-limiting disability. For the purpose of the cohort being representative of the general older population, a past history of any cancer, including CRC, was not an exclusion criterion from ASPREE,^15^ but eligible participants were expected to survive at least 5 years. Following the cessation of trial medication (June 12, 2017), longitudinal observational follow-up continued through the ASPREE-eXTension (ASPREE-XT) study, via continued 6 monthly follow-up, including face-to-face annual visits, and adjudicated clinical event outcomes.^16^ For the purposes of this analysis, ASPREE participants were followed from randomization until the fourth annual visit of the observational phase (utilizing the “ASPREE-XT04” longitudinal dataset), spanning a median of 8.4 years (IQR: 7.2-9.6).

### Ascertainment of NA-NSAID use

The use of concomitant medications (ie, prescription medication and regular [defined as use at least once per week for a minimum of 4 weeks] use of aspirin and NA-NSAIDs) was collected directly from all ASPREE participants at their baseline visit and every subsequent annual visit, by asking participants to bring all their medications to their visit. Additionally, concomitant medication use was established by review of the participant’s medical record. The medication names were processed using artificial intelligence to produce a list of probable Anatomical Therapeutic Chemical (ATC) medication codes, that were reviewed and recorded by 2 staff members. Discordant cases were resolved by consensus. NA-NSAID use at baseline is defined as a binary “Yes” or “No” variable. A participant is defined as an NA-NSAID user at baseline if, at their baseline visit, they reported any medication use with an ATC code starting with “M01A” (the code to classify anti-inflammatory and anti-rheumatic products, non-steroids), excluding “M01AX05” (glucosamine) and “M01AX25” (chondroitin).

The Pharmaceutical Benefits Scheme (PBS) and Medicare Benefits Schedule (MBS) are part of a universal health care system in Australia ensuring that everyone has access to essential medical care. Data from ASPREE has been linked with the Australian PBS/MBS to produce the “ASPREE MBS-PBS Linkage dataset” (version 2), covering the timeframe from randomization to the end of the RCT in June 2017. The MBS/PBS dataset contains prescription medication information, identifiable by ATC code, and supply date, for 13 726 Australian participants (Figure S1). We quantified the PBS-NA-NSAID use recorded in the PBS (as prescription supplied to the recipient) over a 2-year period post-randomization (ensuring the same exposure time period was assessed for all participants) based on the number of prescription supplies, into 3 categories: “None,” if no supplies were recorded, “Light” (indicating occasional or infrequent use), if there was at least 1 but not more than 4 supplies of medications with ATC code starting with “M01A”; and “High” (indicating frequent use), if there were 5 or more supplies. Data regarding dose and duration of NA-NSAIDs was not collected in the ASPREE study. The choice of the cut-off for the number of supplies, while arbitrary, was guided by inspection of the distribution of number of NA-NSAID supplies recorded in the PBS dataset among PBS-NA-NSAID users, at the point of which supply rate plateaued (Figure S2). The categorization as Light or High was made before any analysis of the impact on CRC incidence.

### Ascertainment of CRC outcomes

The primary outcome of this analysis was CRC incidence. Cancer and cancer type (primary anatomical location) were captured by self-report and/or medical record review, and validated by an expert clinical panel utilizing evidentiary documents, including histopathology, specialist letters and imaging.^1^ CRC was defined as any non-metastatic or metastatic solid tumor originating in the colon, caecum, sigmoid or rectum.

### Statistical analysis

The ASPREE-XT04 longitudinal dataset and the ASPREE-MBS/PBS Linkage dataset (version 2) were used in the analysis. Two separate analyses are presented: (1) of all randomized ASPREE participants using the baseline NA-NSAID use (Yes/No); and (2) of the Australian ASPREE participants who consented to access to their PBS data with NA-NSAID exposure categorized as None, Light or High, based on the ASPREE-MBS/PBS Linkage dataset (Figure S1).

The association between NA-NSAID exposure and CRC incidence was investigated using Cox proportional hazards (PH) models with NA-NSAID use as a main variable of interest, adjusted for age at randomization, sex, smoking (Never/Former/Current), alcohol consumption (Never/Former/Current), BMI category (underweight <20 kg/m^2^, normal 20-24.9 kg/m^2^, overweight 25-29.9 kg/m^2^, and obese ≥30 kg/m^2^), past cancer history (Yes/No), history of bowel polyps (Yes/No), family history of CRC (Yes/No) and randomization assignment (aspirin vs placebo), with or without the interaction term between NA-NSAID use and randomized treatment. In the absence of the interaction term, the implicit assumption is that the association between NA-NSAIDs and CRC incidence is the same in the aspirin and placebo arms, while with the interaction term, this assumption is relaxed and the association with NA-NSAIDs may differ. In the PBS-NA-NSAID analysis, smoking history, alcohol, BMI category, past cancer history and family history of CRC were updated with the most recent data available (at 2 years post randomisation). The PH assumption was checked using statistical tests of the Schoenfeld residuals and no violation was observed. To compare the risk of CRC incidence over time by NA-NSAID use, the cumulative incidence of CRC, accounting for competing risk of death, was estimated.

All analyses were performed in R (version 4.2.0; R Core Team, 2020). All analyses are exploratory, no multiple testing adjustment was applied and, if shown, *P*-values are for 2-sided tests.

### Ethics

The ASPREE clinical trial (trial registration: Clinicaltrials.gov number, NCT01038583) and observational follow-up were conducted in accordance with the Declaration of Helsinki 2008, the National Health and Medical Research Council Guidelines on Human Experimentation and the International Conferences of Harmonisation guidelines for Good Clinical Practice. The ethics review board at each participating institution approved the ASPREE clinical trial (Monash University Human Research Ethics Committee [HREC]: CF07/3730-2006000745) and the observational follow-up study (Alfred Health HREC, 17Alfred/198). Written informed consent was obtained from each participant for both the ASPREE study and the PBS linkage.

## Results

### Participant characteristics and NA-NSAID use

Among all randomized ASPREE participants, 14% reported NA-NSAID use at baseline. NA-NSAID users were more likely to be obese, have smoked in the past and be pre-frail (Table 1). When further stratified by randomized assignment, the baseline characteristics did not differ between the aspirin and placebo groups (Table S1). The most frequently used NA-NSAID class reported at baseline was oxicams (40%), with meloxicam the most frequently used within this class (37%), followed by the COX-2 inhibitor class (26%), with celecoxib the most frequent of these (25%). The percentage use of non-specific NA-NSAIDs, such as naproxen (9%), ibuprofen (7%), indomethacin (4%) and others, was 22% (Table 2). Approximately 15% of the NA-NSAID users reported voltaren (diclofenac) type prescription medications at baseline. Note that participants may have been taking more than one type of NA-NSAID.

**Table 1.** Baseline characteristics of ASPREE participants by NA-NSAID use at baseline.

		NA-NSAID use at baseline	
	ASPREE	No	Yes
	(*n* = 19 114)	(*n* = 16 401)	(*n* = 2713)
Age category			
65-74 y	11 166 (58%)	9535 (58%)	1631 (60%)
75-79 y	5020 (26%)	4306 (26%)	714 (26%)
80+ y	2928 (15%)	2560 (16%)	368 (14%)
Sex			
Female	10 782 (56%)	9227 (56%)	1555 (57%)
Education^a^			
≤12 y	10 955 (57%)	9354 (57%)	1601 (59%)
13-15 y	3255 (17%)	2827 (17%)	428 (16%)
16+ y	4903 (26%)	4219 (26%)	684 (25%)
BMI (kg/m^2^)^a^			
Mean (SD)	28.1 (4.72)	27.9 (4.67)	29.1 (4.90)
BMI category^a^			
Normal (20-24.9 kg/m^2^)	4603 (24%)	4126 (25%)	477 (18%)
Underweight (<20 kg/m^2^)	361 (2%)	333 (2%)	28 (1%)
Overweight (25-29.9 kg/m^2^)	8452 (44%)	7257 (44%)	1195 (44%)
Obese (≥30 kg/m^2^)	5609 (29%)	4610 (28%)	999 (37%)
Smoking history			
Never	10 580 (55%)	9182 (56%)	1398 (52%)
Former	7799 (41%)	6586 (40%)	1213 (45%)
Current	735 (4%)	633 (4%)	102 (4%)
Alcohol			
Never	3336 (17%)	2911 (18%)	425 (16%)
Former	1136 (6%)	984 (6%)	152 (6%)
Current	14 642 (77%)	12 506 (76%)	2136 (79%)
Pre-trial cancer history	3679 (19%)	3206 (20%)	473 (17%)
Pre-trial CRC history^a^	472 (2%)	419 (3%)	53 (2%)
Family CRC history	2863 (15%)	2436 (15%)	427 (16%)
History of bowel polyps	3896 (20%)	3333 (20%)	563 (21%)
Diabetes	2045 (11%)	1768 (11%)	277 (10%)
Hypertension	14 196 (74%)	12 119 (74%)	2077 (77%)
CKD^a^	4733 (25%)	4072 (25%)	661 (24%)
Frailty			
Not frail	11 245 (59%)	9810 (60%)	1435 (53%)
Pre-frail	7447 (39%)	6252 (38%)	1195 (44%)
Frail	422 (2%)	339 (2%)	83 (3%)

Abbreviations: BMI = body mass index; CKD = chronic kidney disease; CRC = colorectal cancer.

a Missing values: 1—Education, 89—BMI, 80—Pre-trial CRC history, 1352—CKD (CKD—defined as eGFR < 60 mL/min/1.73 m^2^ or urinary albumin to creatinine ratio ≥3 mg/mmol; Diabetes defined from self-report or fasting glucose ≥ 7 mmol/L or on treatment for diabetes; Frailty—“pre-frail” included anyone with 1 or 2 out of 5 criteria and “Frail” included anyone with 3 or more of the 5 criteria of the adapted Fried frailty criteria, including body weight, strength, exhaustion, walking speed and physical activity; Hypertension—defined as systolic/diastolic blood pressure [SBP/DBP] of ≥140 and/or ≥90 mmHg and/or self-report of antihypertensive medication use).

**Table 2.** Medication and NA-NSAID class frequency of use among the NA-NSAID users.

NA-NSAID class	NA-NSAID name	% of users among NA-NSAID users in ASPREE at baseline (*n* = 2713)	% of users among NA-NSAID users in Australian participants consented to PBS, over 2 years post-randomization (*n* = 4196)
Oxicam	Meloxicam	37%	44%
	Piroxicam	3%	2%
COX-2 inhibitors	Celecoxib	25%	29%
	Etodolac, nabumetone, etoricoxib	1%	0%
Non-specific NA-NSAIDs	Naproxen	9%	9%
	Ibuprofen	7%	8%
	Indometacin	4%	6%
	Ketoprofen, sulindac, tiaprofenic acid	2%	1%
Voltaren	Diclofenac and combinations	14%	16%
	Aceclofenac	<1%	0%

The PBS data allowed us to distinguish between frequent and occasional use of NA-NSAIDs, determined over a 2-year period (PBS-NA-NSAID use) commencing straight after randomization into the ASPREE RCT. Among Australian ASPREE participants consented to PBS linkage, 31% (4196 out of 13 725) had filled a prescription for NA-NSAIDs at least once over the 2-year period, while 67% had no NA-NSAID use recorded in either the PBS dataset or at baseline. Finally, only 2% had reported the use at baseline but none in the PBS dataset (Table 3). The majority of those in the High (frequent) category of PBS-NA-NSAID users (75%: 1162 out of 1573) also reported NA-NSAID use at baseline, and conversely, the majority of those who reported baseline NA-NSAID use were frequent NA-NSAID users according to the PBS data (60%: 1162 out of 1980). Those who did not report NA-NSAID use at baseline but had filled NA-NSAID prescriptions at least once in 2 years were mainly participants who fell into the Light (occasional) PBS-NA-NSAID use category (84%: 2150 out of 2561). The frequencies pattern of NA-NSAIDs prescribed over 2 years post-randomization is similar to baseline (Table 2). As was expected, the characteristics of the High PBS-NA-NSAID users were similar to the baseline NA-NSAID users; and were more likely to be obese, to have smoked in the past and be pre-frail (Table S2). There were no major differences in the distribution of characteristics (smoking history, alcohol, BMI category, past cancer history, and family history of CRC) as at year 2 post-randomization used in the PBS-NA-NSAID analysis, compared to the baseline distribution (Table S3).

**Table 3.** Concordance between the quantification of PBS-NA-NSAID users over 2 years post-randomization in Australian ASPREE participants consented to PBS (*n* = 13 725) and their reported NA-NSAID use at the baseline.

Number of NA-NSAID users at baseline	Number of PBS-NA-NSAID users, over 2 years post-randomization^a^		
	None (*n* = 9529)	Light (≤4 supplies) (*n* = 2623)	High (5+ supplies) (*n* = 1573)
No (*n* = 11 745)	9184 (67%)	2150 (16%)	411 (3%)
Yes (*n* = 1980)	345 (2%)	473 (3%)	1162 (9%)

a Percentages in the table are out of a total of 13 725, to show concordance, and are not either by row or by column.

### NA-NSAID use and CRC incidence

**Figure 1.**
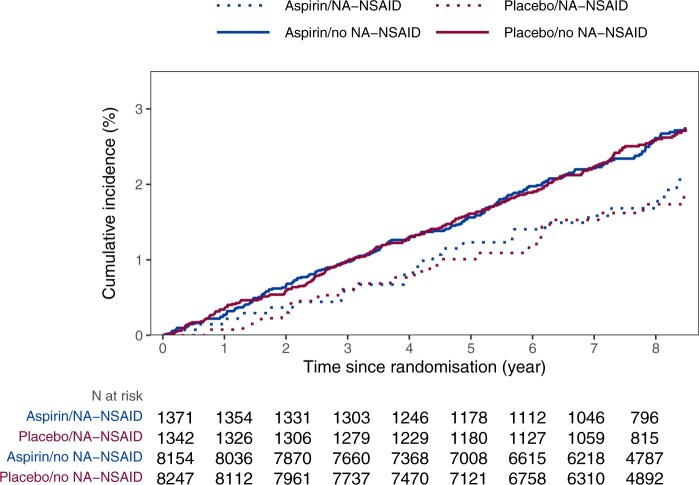
Cumulative incidence of CRC by NA-NSAID use at baseline and randomized treatment.

Over the total follow-up of 150 620 person-years from randomization of all ASPREE participants, 476 cases of CRC were documented. The CRC event rate among the NA-NSAID users was 2.4/1000 person-years compared to 3.3/1000 person-years among non-users (Table 4). NA-NSAID use was associated with a reduced CRC incidence (HR^NA-NSAID use: Yes vs No^ = 0.74, 95% CI = 0.56 to 0.98). There was no evidence to suggest that the association between NA-NSAIDs and CRC incidence is modified by concomitant aspirin use (Table S4). Indeed, in the placebo group, the HR^NA-NSAID use: Yes vs No^ was estimated to be 0.71 (95% CI = 0.47 to 1.08) and in the Aspirin group, the HR^NA-NSAID use: Yes vs No^ was 0.77 (95% CI = 0.51 to 1.14) with a *P*-value for the interaction term of 0.81. The cumulative incidence of CRC by NA-NSAID use at baseline and randomized treatment over an 8-year period shows that CRC incidence over time is lower in NA-NSAID users compared to non-users, and that the association between NA-NSAIDs and CRC was not altered by additional use of aspirin (Figure 1).

**Table 4.** The effect of NA-NSAID use on CRC incidence as assessed by Cox PH models in ASPREE-XT.

Analysis	Exposure ascertainment	NA-NSAID exposure	Total follow-up person-years	No. of pts	CRC event number	Event rates/1000 py (95% CI)	HR (95% CI)^a^
^b^ASPREE-XT (Follow up for CRC events from randomization [baseline], median 8.4 y [IQR: 7.2-9.6])	NA-NSAID use at baseline	No	129 059	16 326	424	3.3 (3.0 to 3.6)	Reference
		Yes	21 561	2699	52	2.4 (1.8 to 3.2)	0.74 (0.56 to 0.98)
^c^ASPREE-XT (Follow up for CRC events from year 2, median 6.4 y [IQR: 5.2-7.6])	PBS-NA-NSAID use over 2 years post-randomization	None	62 061	9445	213	3.4 (3.0 to 3.9)	Reference
		Light (≤4 supplies over 2 years)	16 984	2597	49	2.9 (2.1 to 3.8)	0.86 (0.63 to 1.18)
		High (5+ supplies over 2 years)	10 182	1559	19	1.9 (1.1 to 2.9)	0.52 (0.33 to 0.83)

Abbreviation: py = person years.

a Hazard ratios are adjusted for age, sex, BMI category, smoking history, alcohol, past cancer history, family CRC (parents/sibling) history, history of bowel polyps and randomized treatment, without the interaction term between NA-NSAID use and randomized treatment. In the PBS-NA-NSAID analysis, the covariates were updated with the most recent information at 2 years post-randomization.

b CRC events ascertained from randomization (baseline) for ASPREE-XT participants.

c CRC events as of year 2 post-randomization, following NA-NSAID exposure ascertainment, using the ASPREE-PBS dataset (PBS-NA-NSAID use).

**Figure 2.**
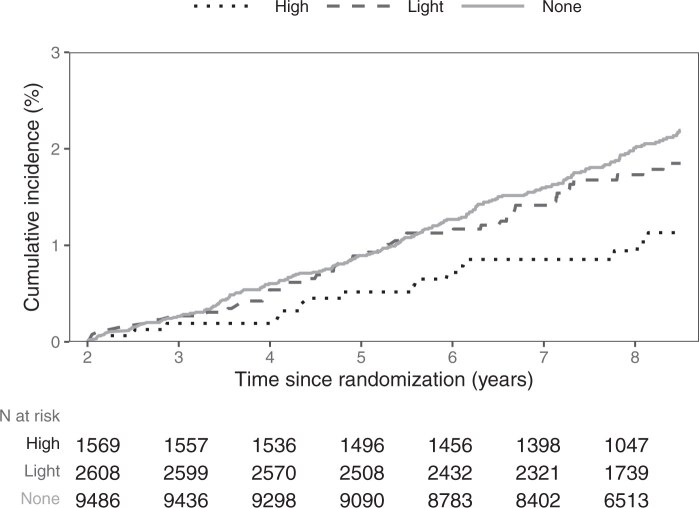
Cumulative incidence of CRC by PBS-NA-NSAID use category, with a time reset at 2-years post-randomization.

Over the total follow-up of 89 227 person-years from 2 years post-randomization onwards, among the Australian ASPREE participants who consented to use of PBS data and did not have CRC incidence within the 2-year PBS-NA-NSAID exposure ascertainment period, 281 participants were diagnosed with a CRC. The CRC event rate among participants with no recorded use of NA-NSAIDs in PBS (“None” category) was the highest with 3.4/1000 person-years compared to 2.9 and 1.9/1000 person-years in the “Light” and “High” PBS-NA-NSAID use categories, respectively (Table 4). The frequent use of NA-NSAIDs (“High” category) appears to be associated with reduced CRC incidence with a HR^PBS-NA-NSAID use: High vs None^ of 0.52 (95% CI = 0.32 to 0.83) when compared to non-users. The occasional use of NA-NSAIDs (“Light” category) compared to no recorded use in the PBS dataset, did not influence CRC risk (HR^PBS-NA-NSAID use: Light vs None^ of 0.86, 95% CI = 0.63 to 1.18). While CRC incidence is lower in those with more frequent (high) NA-NSAID and aspirin use than aspirin only users, there is no evidence to suggest that the association between NA-NSAID use and CRC incidence is modified by aspirin, as demonstrated by the interaction analysis (*P* = .42; Table S4). Cumulative incidence of CRC by PBS-NA-NSAID categories also confirms the beneficial association between NA-NSAID use on CRC incidence (Figure 2). We explored if specific NA-NSAID classes differentially associated with CRC incidence (Table S5), noting that the COX-2 inhibitors and oxicams were the most frequently used class in our cohort of older adults. Oxicam, voltaren, and non-specific NA-NSAIDs appear to be associated with a lower CRC risk, as indicated by the point estimates (however, statistical significance was not reached), while users of COX-2 inhibitors have a similar risk of CRC as NA-NSAID-non-users.

## Discussion

In this secondary analysis of community-dwelling healthy older adults participating in the ASPREE study, we found that NA-NSAID use was associated with a decreased risk of developing CRC. This effect was independent of the use of aspirin, in keeping with evidence from earlier studies^1^^,^^13^^,^^17^ and is in the context of the overall findings of the ASPREE RCT, which demonstrated that there was no significant difference between aspirin and placebo groups in terms of incidence of all cancers, including CRC.^1^ Based on analyses of other population-based studies, particular subgroups of healthy individuals, including adults aged >40 years,^11^ adults >50 years,^18^ and postmenopausal women,^10^ may benefit from NA-NSAIDs for CRC prevention, as well as an unselected population.^7^^,^^8^^,^^19^ Our findings suggest that adults over the age of 70 are another subgroup who may benefit from NA-NSAIDs in preventing CRC incidence. However, further investigation is needed to explore NA-NSAIDs safety and toxicity, in light of the well-described cardiovascular and gastrointestinal bleeding risks, which are increased in an older population (especially when in combination with aspirin). This is particularly important given that beneficial effects were associated with higher frequency of NA-NSAID use.

Shebl et al. have previously demonstrated that the combined use of aspirin and NA-NSAIDs in healthy adults (mean age 62.9 years, SD 5.3) was associated with a reduced risk of cancers, including CRC, compared to non-use of either aspirin or NA-NSAID.^20^ In contrast, a case control study of healthy adults aged 40-75 years observed a reduced risk of pre-cancerous colon adenomas in individuals regularly using either aspirin or NA-NSAIDs for at least 3 days a week over a period of one year, but no additional statistically significant difference in patients reporting both regular aspirin and NA-NSAID use, compared to use of a single agent.^13^ Clearly, further exploration is required to better understand the impact, if any, of concurrent aspirin and NA-NSAID use on subsequent CRC-related outcomes. In our study, there was no significant interaction between NA-NSAID use and aspirin, suggesting that any beneficial effects of NA-NSAIDs on CRC incidence are independent of aspirin.

Both NA-NSAIDs and aspirin block the metabolism of arachidonic into prostaglandin H2, via inhibition of COX-1 and COX-2.^21^ COX-1 inhibition leads to a reduction in cytoprotective prostaglandins and anti-platelet activity, while COX-2 inhibition leads to decreased production of vasodilator prostaglandins and a decrease in the pro-inflammatory state. NSAIDs are commonly classified as either non-selective, which includes aspirin (ie, inhibiting both COX-1 and COX-2), or COX-2 selective.^5^ Notably, aspirin irreversibly inactivates both COX-1 and COX-2 through non-competitive binding, but is more potent at inhibiting COX-1. In contrast, NA-NSAIDs reversibly inhibit COX enzymes through competitive binding. The cancer preventive benefits of COX-2 inhibitors are likely mediated through suppression of chronic inflammation, which is a known contributor to the development of cancers such as CRC.^6^ However, while COX inhibition is presumed to be the main mechanism of antineoplastic activity of NSAIDs, COX-independent mechanisms, such as inducing cell apoptosis and inhibition of angiogenesis, are also thought to play a role.^22^

Importantly, more frequent use of NA-NSAIDs was associated with a reduced risk of CRC incidence, whereas infrequent use (“Light” category) or no use was not. This is in keeping with an earlier population-based, nested case control study (median age 72 years) demonstrating that prolonged NSAID use (>25 prescriptions in 13-48 months) was associated with a greater risk reduction in CRC incidence.^8^ In addition, while 2 different methods of ascertaining NA-NSAID exposure were utilized in our analyses (initiating multi-year low-dose aspirin in NA-NSAID users [NA-NSAID at baseline] and simultaneous multi-year NA-NSAID use with low-dose aspirin [PBS-NA-NSAID]), both methods led to similar associations with CRC. The follow-up duration of 8.4 years (and 6.4 years in the PBS subset) in this study is similar to others reporting a beneficial effect of NSAID use on CRC incidence, with a median follow-up of up to 8 years.^8^^,^^23^ In our study, we analyzed the frequency of NA-NSAID prescriptions that were filled and supplied to the individual, but not the dose and duration of NA-NSAIDs used (this data was not collected by the ASPREE study). The importance of duration and dose of NSAIDs is highlighted in previous studies exploring NSAIDs in CRC chemoprevention in high risk populations such as Lynch syndrome,^24^ as well as in CRC outcomes,^25^ and in healthy adults.^7^ Further analysis of duration and dose intensity of NA-NSAID use and impacts on CRC incidence would be of interest.

Strengths of our study include the use of prospectively collected data from a very large cohort of older adults, with additional national data linkage. Comprehensive phenotypic data was regularly collected, and the use of blinded expert reviewers to ascertain cancer incidence events minimizes the risk of bias and incorrect classifications. Limitations of our study include that ASPREE involved a cohort of initially healthy older adults, more so than the general population, and thus, any conclusions from this study are only directly applicable to this particular population subset, rather than the wider population. Secondly, our ascertainment of NA-NSAID exposure using PBS data was limited to patients receiving prescribed NA-NSAIDs, and did not include over-the-counter (OTC, without prescription) use of NA-NSAIDs, which are widely available in Australia. However, regular (defined as at least once per week for a minimum of 4 weeks) use of OTC NA-NSAIDs was captured annually from participants or via medical record review.

Analysis of other health-related outcomes in participants who had concurrent aspirin and NA-NSAID use in ASPREE may provide further insights. While physical exercise and diet data were not available, we acknowledge that our analysis would have been strengthened by their inclusion as covariates. Additionally, it was not possible to ascertain indications for NA-NSAID use and whether such concomitant medical conditions may confound our findings. NSAIDs are typically used in individuals without important comorbidities such as significant cardiovascular or renal disease. While baseline clinical factors were typically well-balanced between NA-NSAID users and non-users, the NA-NSAIDs users were more likely to be frail or pre-frail, former smokers and have a higher BMI, which may impact our findings. Finally, the length of follow-up may not be long enough for all CRC incidences to be observed, particularly given that CRC typically takes around 10 years to develop, and as such, this analysis would benefit from longer follow-up.

## Conclusions

In otherwise healthy older adults aged predominantly over 70 years, this post hoc analysis of the ASPREE population demonstrates that NA-NSAID use is associated with a reduction in CRC incidence, which was not modified by concurrent aspirin use. Further analysis of dose intensity and duration of NA-NSAID use on CRC incidence would be of interest.

## Supplementary Material

djaf145_Supplementary_Data

## Data Availability

The data underlying this article will be made available on reasonable request, addressed to the ASPREE Access Management System (AMS): AMS@monash.edu.
