# Supplementary material for: Association of non-steroidal anti-inflammatory medications and aspirin with colorectal cancer incidence in older adults
Source: J Natl Cancer Inst. 2025 Jun 14;117(9):1875–82. doi: 10.1093/jnci/djaf145 (PMC12415954; doi:10.1093/jnci/djaf145)

Supplementary Table 1. Baseline characteristics of ASPREE participants by NA-NSAID use at baseline and study treatment (Aspirin/Placebo).

	NA-NSAID use at baseline			
	No (N = 16,401)		Yes (N = 2,713)	
	Placebo (N = 8,247)	Aspirin (N = 8,154)	Placebo (N = 1,342)	Aspirin (N = 1,371)
Age category				
65-74yo	4839 (59 %)	4696 (58 %)	799 (60 %)	832 (61 %)
75-79yo	2122 (26 %)	2184 (27 %)	366 (27 %)	348 (25 %)
80+yo	1286 (16 %)	1274 (16 %)	177 (13 %)	191 (14 %)
Sex				
Female	4636 (56 %)	4591 (56 %)	773 (58 %)	782 (57 %)
Country				
AUS	7174 (87 %)	7100 (87 %)	1207 (90 %)	1222 (89 %)
US	1073 (13 %)	1054 (13 %)	135 (10 %)	149 (11 %)
Education				
≤12yr	4683 (57 %)	4671 (57 %)	800 (60 %)	801 (58 %)
13-15yr	1408 (17 %)	1419 (17 %)	210 (16 %)	218 (16 %)
16+yrs	2156 (26 %)	2063 (25 %)	332 (25 %)	352 (26 %)
BMI (kg/m2)				
Mean (SD)	27.9 (4.61)	28.0 (4.73)	29.2 (4.92)	29.0 (4.88)
BMI category				
Normal (20 – 24.9 kg/m^2)	2065 (25 %)	2061 (25 %)	222 (17 %)	255 (19 %)
Underweight (<20 kg/m^2)	166 (2 %)	167 (2 %)	10 (1 %)	18 (1 %)
Overweight (25 –2 9.9 kg/m^2)	3685 (45 %)	3572 (44 %)	578 (43 %)	617 (45 %)
Obese (≥30 kg/m^2)	2292 (28 %)	2318 (28 %)	525 (39 %)	474 (35 %)
Smoking history				
Never	4607 (56 %)	4575 (56 %)	709 (53 %)	689 (50 %)
Former	3306 (40 %)	3280 (40 %)	584 (44 %)	629 (46 %)
Current	334 (4 %)	299 (4 %)	49 (4 %)	53 (4 %)
Alcohol				
Never	1466 (18 %)	1445 (18 %)	220 (16 %)	205 (15 %)
Former	500 (6 %)	484 (6 %)	70 (5 %)	82 (6 %)
Current	6281 (76 %)	6225 (76 %)	1052 (78 %)	1084 (79 %)
Past cancer history	1618 (20 %)	1588 (19 %)	221 (16 %)	252 (18 %)
Past CRC history	212 (3 %)	207 (3 %)	23 (2 %)	30 (2 %)
Family CRC history	1227 (15 %)	1209 (15 %)	207 (15 %)	220 (16 %)
History of bowel polyps	1724 (21 %)	1609 (20 %)	276 (21 %)	287 (21 %)
Diabetes	885 (11 %)	883 (11 %)	136 (10 %)	141 (10 %)
Hypertension	6112 (74 %)	6007 (74 %)	1027 (77 %)	1050 (77 %)
CKD	2037 (25 %)	2035 (25 %)	326 (24 %)	335 (24 %)
Frailty				
Not frail	4918 (60 %)	4892 (60 %)	721 (54 %)	714 (52 %)
Pre-frail	3158 (38 %)	3094 (38 %)	584 (44 %)	611 (45 %)
Frail	171 (2 %)	168 (2 %)	37 (3 %)	46 (3 %)

Abbreviations: BMI, Body mass Index; CRC, Colorectal cancer; CKD, chronic kideny disease.
(CKD – defined as eGFR < 60mL/min/1.73m^2 or urinary albumin to creatinine ratio ≥3 mg/mmol; Diabetes defined from self-report or fasting glucose ≥ 7mmol/L or on treatment for diabetes; Frailty – 'pre-frail' included anyone with 1 or 2 criteria and 'Frail' included anyone with 3 or more criteria of the adapted Fried frailty criteria, including body weight, strength, exhaustion, walking speed and physical activity; Hypertension – defined as systolic/diastolic blood pressure [SBP/DBP] of ≥140 and/or ≥90 mmHg and/or self-report of antihypertensive medication use).

Supplementary Table 2. Baseline characteristics of ASPREE participants, Australian ASPREE participants consented to PBS and Australian ASPREE participants PBS-NA-NSAID categories.

	ASPREE (N=19,114)	Australian ASPREE participants consented to PBS (N=13,725)	PBS-NA-NSAID		
			None (N=9,529)	Light (≤ 4 supplies) (N=2,623)	High (5+ supplies) (N=1,573)
Age category					
65-74yo	11166 (58 %)	8324 (61 %)	5706 (60 %)	1643 (63 %)	975 (62 %)
75-79yo	5020 (26 %)	3580 (26 %)	2469 (26 %)	692 (26 %)	419 (27 %)
80+yo	2928 (15 %)	1821 (13 %)	1354 (14 %)	288 (11 %)	179 (11 %)
Sex					
Female	10782 (56 %)	7458 (54 %)	5144 (54 %)	1404 (54 %)	910 (58 %)
Country					
AUS	16703 (87 %)	13725 (100 %)	9529 (100 %)	2623 (100 %)	1573 (100 %)
US	2411 (13 %)	0 (0 %)	0 (0 %)	0 (0 %)	0 (0 %)
Education					
≤12yr	10955 (57 %)	8193 (60 %)	5665 (59 %)	1565 (60 %)	963 (61 %)
13-15yr	3255 (17 %)	2126 (15 %)	1481 (16 %)	418 (16 %)	227 (14 %)
16+yrs	4903 (26 %)	3405 (25 %)	2382 (25 %)	640 (24 %)	383 (24 %)
BMI (kg/m2)					
Mean (SD)	28.1 (4.72)	28.0 (4.54)	27.7 (4.43)	28.2 (4.50)	29.4 (4.93)
BMI category					
Normal (20 – 24.9 kg/m^2)	4603 (24 %)	3306 (24 %)	2460 (26 %)	588 (22 %)	258 (16 %)
Underweight (<20 kg/m^2)	361 (2 %)	218 (2 %)	170 (2 %)	38 (1 %)	10 (1 %)
Overweight (25 –2 9.9 kg/m^2)	8452 (44 %)	6235 (45 %)	4355 (46 %)	1215 (46 %)	665 (42 %)
Obese (≥30 kg/m^2)	5609 (29 %)	3903 (28 %)	2503 (26 %)	770 (29 %)	630 (40 %)
Smoking history					
Never	10580 (55 %)	7733 (56 %)	5494 (58 %)	1465 (56 %)	774 (49 %)
Former	7799 (41 %)	5590 (41 %)	3764 (40 %)	1081 (41 %)	745 (47 %)
Current	735 (4 %)	402 (3 %)	271 (3 %)	77 (3 %)	54 (3 %)
Alcohol					
Never	3336 (17 %)	2172 (16 %)	1554 (16 %)	379 (14 %)	239 (15 %)
Former	1136 (6 %)	615 (4 %)	438 (5 %)	111 (4 %)	66 (4 %)
Current	14642 (77 %)	10938 (80 %)	7537 (79 %)	2133 (81 %)	1268 (81 %)
Past cancer history	3679 (19 %)	2656 (19 %)	1890 (20 %)	493 (19 %)	273 (17 %)
Past CRC history	472 (2 %)	360 (3 %)	256 (3 %)	68 (3 %)	36 (2 %)
Family CRC history	2863 (15 %)	2116 (15 %)	1450 (15 %)	406 (15 %)	260 (17 %)
History of bowel polyps	3896 (20 %)	2750 (20 %)	1847 (19 %)	568 (22 %)	335 (21 %)
Diabetes	2045 (11 %)	1290 (9 %)	857 (9 %)	264 (10 %)	169 (11 %)
Hypertension	14196 (74 %)	10219 (74 %)	7109 (75 %)	1889 (72 %)	1221 (78 %)
CKD	4733 (25 %)	3151 (23 %)	2219 (23 %)	562 (21 %)	370 (24 %)
Frailty					
Not frail	11245 (59 %)	8805 (64 %)	6216 (65 %)	1676 (64 %)	913 (58 %)
Pre-frail	7447 (39 %)	4728 (34 %)	3185 (33 %)	913 (35 %)	630 (40 %)
Frail	422 (2 %)	192 (1 %)	128 (1 %)	34 (1 %)	30 (2 %)

Abbreviations: BMI, Body mass Index; CRC, Colorectal cancer; CKD, chronic kideny disease.
(CKD – defined as eGFR < 60mL/min/1.73m^2 or urinary albumin to creatinine ratio ≥3 mg/mmol; Diabetes defined from self-report or fasting glucose ≥ 7mmol/L or on treatment for diabetes; Frailty – 'pre-frail' included anyone with 1 or 2 criteria and 'Frail' included anyone with 3 or more criteria of the adapted Fried frailty criteria, including body

weight, strength, exhaustion, walking speed and physical activity; Hypertension – defined as systolic/diastolic blood pressure [SBP/DBP] of ≥140 and/or ≥90 mmHg and/or self-report of antihypertensive medication use).

Supplementary Table 3. The comparison of smoking history, alcohol consumption, BMI category, past personal CRC history and family history of CRC, updated at year 2 post-randomization for the PBS-NA-NSAID analysis versus these characterisitics as at baseline.

Smoking history		*Year 2 post-randomization*			
		Never	Former	Current	
Baseline	Never	7,661	0	1	
	Former	0	5,495	48	
	Current	0	96	300	
Alcohol					
		Never	Former	Current	
Baseline	Never	1,865	70	210	
	Former	0	480	131	
	Current	0	925	9,920	
BMI category					
		Normal	Underweight	Overweight	Obese
Baseline	Normal	2,781	92	417	2
	Underweight	46	170	0	0
	Overweight	552	0	5,256	397
	Obese	6	0	537	3,345
Past personal cancer history					
		No	Yes		
			Pre-trial	In-trial[a]	
Baseline	No	10,718	0	254	
	Yes	0	2,629	0	
Family history of CRC					
		No	Yes		
Baseline	No	11,418	78		
	Yes	0	2,105		

[a] Any cancer except CRC within 2 years post-randomization. Participants with an in-trial CRC during the 2 years post-randomization were excluded from the PBS-NA-NSAID analysis.

Supplementary Table 4. The effect of NA-NSAID use on CRC incidence as assessed by Cox PH models in ASPREE-XT with the interaction term between NA-NSAID use and randomized treatment.

ASPREE-XT [Follow up for CRC events from randomization, median 8.4 years (IQR: 7.2 – 9.6)]						
NA-NSAID use at baseline	Total follow-up person-years	N of pts	CRC events	Event rates/1000 py (95% CI)	HR (95%CI)[a]	P-value
	Placebo					
No	65039	8208	213	3.3 (2.8 - 3.7)	Reference	
Yes	10746	1335	25	2.3 (1.5 - 3.4)	0.71 (0.47 - 1.08)	0.11
	Aspirin					
No	64020	8118	211	3.3 (2.9 - 3.8)	Reference	
Yes	10816	1364	27	2.5 (1.6 - 3.6)	0.77 (0.51 - 1.14)	0.19
Interaction term					Ratio of HRs (95%CI)	P-value
	HR Yes vs No in Aspirin		HR Yes vs No in Placebo		1.08 (0.60 - 1.91)	0.81
ASPREE-XT [Follow-up for CRC events from year 2 (post NA-NSAID ascertainment period), median 6.4 years (IQR: 5.2 – 7.6)]						
PBS-NA-NSAID use over 2 years post-randomization	Total follow-up person-years	N of pts	CRC events	Event rates/1000 py (95% CI)	HR (95%CI)[a]	P-value
	Placebo					
None	31445	4774	111	3.5 (2.9 - 4.3)	Reference	
Light (≤ 4 supplies in 2 yrs)	8373	1277	27	3.2 (2.1 - 4.7)	0.94 (0.61 - 1.43)	0.76
High (5+ supplies in 2 yrs)	5257	809	12	2.3 (1.2 - 4.0)	0.61 (0.34 - 1.11)	0.11
	Aspirin					
None	30616	4671	102	3.3 (2.7 - 4.0)	Reference	
Light (≤ 4 supplies in 2 yrs)	8611	1320	22	2.6 (1.6 - 3.9)	0.79 (0.50 - 1.25)	0.31
High (5+ supplies in 2 yrs)	4925	750	7	1.4 (0.6 - 2.9)	0.41 (0.19 - 0.89)	0.02
Interaction term					Ratio of HRs (95%CI)	P-value
	HR Light vs None in Aspirin		HR Light vs None in Placebo		0.84 (0.45 - 1.57)	0.58
	HR High vs None in Aspirin		HR High vs None in Placebo		0.67 (0.25 - 1.77)	0.42

[a]Hazard ratios are from the Cox PH model adjusted for age, sex, BMI category, smoking history, alcohol, past cancer history and family CRC (parents/sibling) history, history of bowel polyps and randomized treatment with the interaction term between NA-NSAID use and randomized treatment. In the PBS-NA-NSAID analysis, the covariates were updated with the most recent information prior 2 years post-randomization

Supplementary Table 5. The effect of NA-NSAID use by class on CRC incidence, as assessed by Cox PH models in ASPREE-XT.

[a]ASPREE-XT [Follow up for CRC events from randomization (baseline), median 8.4 years (IQR: 7.2 – 9.6)].					
NA-NSAID use by class at baseline	Total follow-up person-years (Total = 150,341)	N of pts[b] (Total = 19,025)	CRC event number (Total = 476)	Event rates/1000 py (95% CI)	HR (95%CI)[c]
None	129,059	16,326	424	3.3 (3.0 – 3.6)	Reference
Oxicam	8,534	1,064	20	2.3 (1.4 – 3.6)	0.72 (0.46 – 1.13)
Cox-2 inhibitors	5,415	685	17	3.1 (1.8 – 5.0)	0.96 (0.59 – 1.56)
Non-specific NSAIDs	4,399	556	9	2.0 (0.9 – 3.9)	0.62 (0.32 – 1.20)
Voltaren	2,934	361	6	2.0 (0.8 – 4.5)	0.63 (0.28 – 1.41)
[d]ASPREE-XT [Follow up for CRC events from year 2, median 6.4 years (IQR: 5.2 – 7.6)]					
PBS-NA-NSAID use by class over 2 years post-randomization	Total follow-up person-years (Total = 85,953)	N of pts[e] (Total = 13,096)	CRC event number (Total = 275)	Event rates/1000 py (95% CI)	HR (95%CI)[c]
None	62,061	9,445	213	3.4 (3.0 – 3.9)	Reference
Oxicam	10,071	1,543	25	2.5 (1.6 – 3.7)	0.72 (0.47 – 1.09)
Cox-2 inhibitors	6,225	949	18	2.9 (1.7 – 4.6)	0.85 (0.52 – 1.37)
Non-specific NSAIDs	4,563	701	11	2.4 (1.2 – 4.3)	0.70 (0.38 – 1.29)
Voltaren	3,033	458	8	2.6 (1.1 – 5.2)	0.79 (0.39 – 1.61)

[a]CRC events ascertained from randomisation (baseline) for ASPREE-XT participants

[b] 33 participants were excluded from the analysis because they reported the use of two types of NA-NSAIDs.
[c]Hazard ratios are adjusted for age, sex, BMI category, smoking history, alcohol, past cancer history, family CRC (parents/sibling) history, history of bowel polyps and randomized treatment. In the PBS-NA-NSAID analysis, the covariates were updated with the most recent information prior 2 years post-randomization.
[d]CRC events as of year 2 post randomisation, following NA-NSAID exposure ascertainment and excluding those with an in-trial CRC diagnosis during the 2-year NA-NSAID use ascertainment period, using the ASPREE-PBS dataset (PBS-NA-NSAID use).
[e]505 participants were excluded from the analysis as they had more than one type of NA-NSAIDs recorded in PBS in the first 2 years post-randomization

Supplementary Figure 1. Participant selection flow chart.
Grey boxes provide reasons for exclusion from the analysis with PBS-NA-NSAIDs.

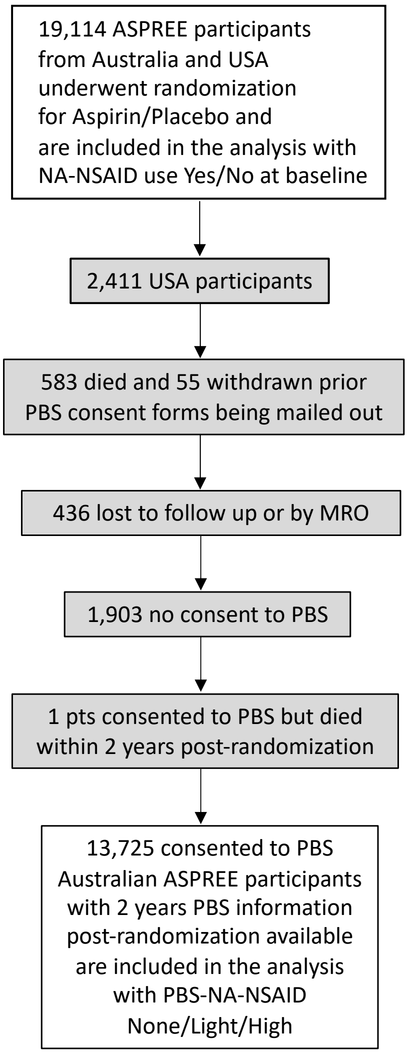

Supplementary Figure 2. Distribution of NA-NSAID PBS supplies over 2 years post-randomization for Australian ASPREE participants consented to PBS and who had at least one supply recorded (N = 4,196).

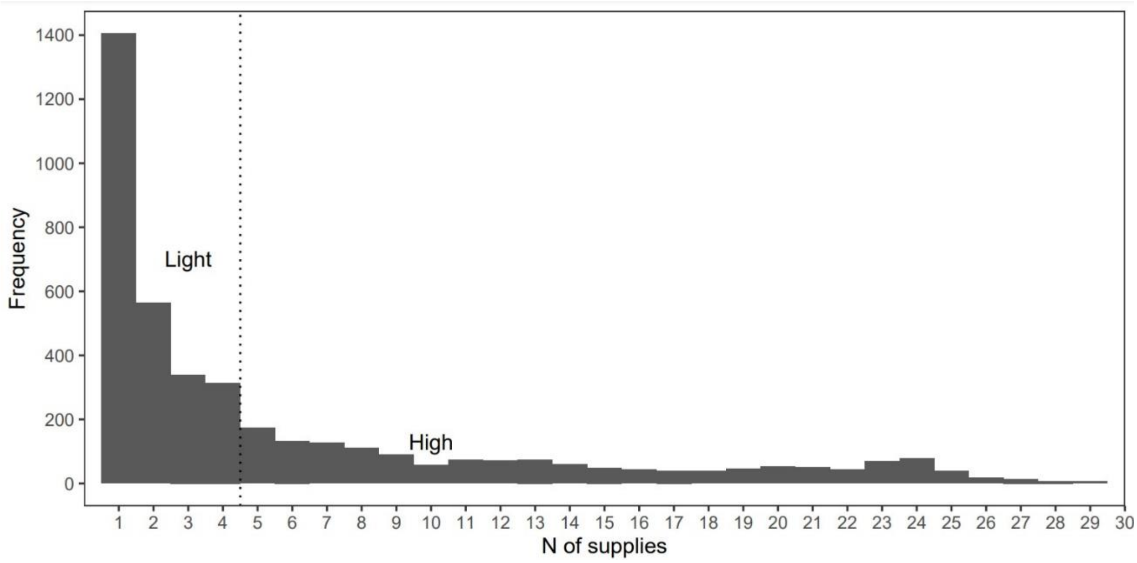

Supplement: djaf145_Supplementary_Data [file djaf145_supplementary_data.pdf]
